# Combining calls from multiple somatic mutation-callers

**DOI:** 10.1186/1471-2105-15-154

**Published:** 2014-05-21

**Authors:** Su Yeon Kim, Laurent Jacob, Terence P Speed

**Affiliations:** 1 Department of Statistics, University of California at Berkeley, Berkeley CA 94720, USA; 2 Laboratoire de Biométrie et Biologie Evolutive, Université de Lyon, Université Lyon 1, CNRS, INRA, UMR5558 Villeurbanne, France; 3, Walter and Eliza Hall Institute of Medical Research and the University of Melbourne, Parkville, Victoria, Australia

**Keywords:** Cancer genome, Somatic mutation-calling, Combining calls, Stacking

## Abstract

**Background:**

Accurate somatic mutation-calling is essential for insightful mutation analyses in cancer studies. Several mutation-callers are publicly available and more are likely to appear. Nonetheless, mutation-calling is still challenging and there is unlikely to be one established caller that systematically outperforms all others. Therefore, fully utilizing multiple callers can be a powerful way to construct a list of final calls for one’s research.

**Results:**

Using a set of mutations from multiple callers that are impartially validated, we present a statistical approach for building a combined caller, which can be applied to combine calls in a wider dataset generated using a similar protocol. Using the mutation outputs and the validation data from The Cancer Genome Atlas endometrial study (6,746 sites), we demonstrate how to build a statistical model that predicts the probability of each call being a somatic mutation, based on the detection status of multiple callers and a few associated features.

**Conclusion:**

The approach allows us to build a combined caller across the full range of stringency levels, which outperforms all of the individual callers.

## Background

Somatic mutations are genetic changes that occur in somatic cells after conception. Cancer is driven by such somatic alterations, and thus cataloging somatic mutations is essential to understand the genetic bases of cancer development. With the burst of high-throughput sequencing data generated in recent years, extensive efforts have been made towards accurate somatic mutation-calling. Many calling algorithms are now publicly available, including Shimmer [1], MuTect [2], Strelka [3], MutationSeq [4], JointSNVMix [5], and SomaticSniper [6]. Additional in-house callers are likely to be under development for on-going studies. Nonetheless, many challenges remain to be addressed, for example, removing artifactual variants from multiple sources, detecting rare variants in highly heterogeneous tumor samples, detecting variants at a shallower sequencing coverage. Every caller will tackle these issues, but different callers are likely to be more successful on some of them and less so on others. As a consequence, finding the single best performing caller is not easy, and perhaps not even feasible.

Having multiple callers on hand, anyone conducting a mutation analysis may want to apply all of the callers to his/her data with the aim of later constructing a list of final calls. In essence, combining calls from multiple callers amounts to developing a strategy to sort the calls to be included as final calls. This can be done effectively if one can systematically assign a confidence measure to be a somatic mutation across the full list. In general, pursuing this goal requires a validation dataset to some extent. For example, the paper by Lower et al. [7] presented a method to prioritize calls from three methods by assigning false discovery rate confidence values, but it requires the independent sequencing of at least one of the tumor or normal samples.

In our work, we are considering a situation in which mutation-calling is done (by multiple callers) for many tumor-normal sequence pairs across a large genomic regions such as whole genome or exome, but only a limited resource is available for validation. For example, in practice, often only a small fraction of detected mutations can be validated or a small subset of regions in a selected list of samples are re-sequenced for evaluation purposes. We aim to build a combined caller, which is learned based on the relatively small validation dataset but can be applied to a wider dataset generated based on a similar protocol.

A large corpus in the statistical literature is dedicated to combining individual learners, see *e.g.* Chapter 16 of [8], however most of them — *e.g.*, boosting, bagging and random forests — are based on building individual learners from descriptors rather than combining outputs of algorithms. *Stacking*[9] was introduced as a mean of combining such outputs. In this paper, we exploit this well established framework to merge the outputs of different callers.

Specifically, we present a statistical approach for combining calls from multiple somatic mutation-callers, when validation is impartially done for all mutations detected by all callers in a selected set of regions or samples. For 194 tumor-normal exome-seq pairs from The Cancer Genome Atlas (TCGA) endometrial study [10], single nucleotide variant (SNV) type mutations (i.e., point mutations) were detected by three somatic mutation-callers. Validation through an independent re-sequencing was impartially done for all the mutations detected from 20 selected patients across the whole exome and for those mutations detected within 243 genes of interest across all 194 patients. We used this data to show how our statistical approach improves against individual callers and naive combination based on caller intersection. We also show that this improvement is maintained when the parameters of the model are estimated on a set of samples or regions different from the ones on which the performance is evaluated.

## Methods

Our aim is to build a combined caller using the mutation outputs generated by multiple callers based on the same paired tumor-normal sequence data (BAMs; [11]), when the mutation calls are impartially validated. For illustration purposes, we assume *K*=3 callers (Caller A, B, and C) are used for mutation-calling. The most basic and key information available in each mutation output is the list of positions detected as point mutations. A mutation output may include additional features such as mutation substitution type, mutation quality score, and perhaps details of filters applied to remove artifactual or low-quality variants. When the raw sequence data are available, genomic features can be computed for each mutation site such as sequencing depth and the variant allele fraction (the fraction of reads carrying the variant allele) for each tumor and normal sample. The more information that is available, the more powerful are the callers that can be constructed.

### Taking intersections or unions

**Figure 1:**
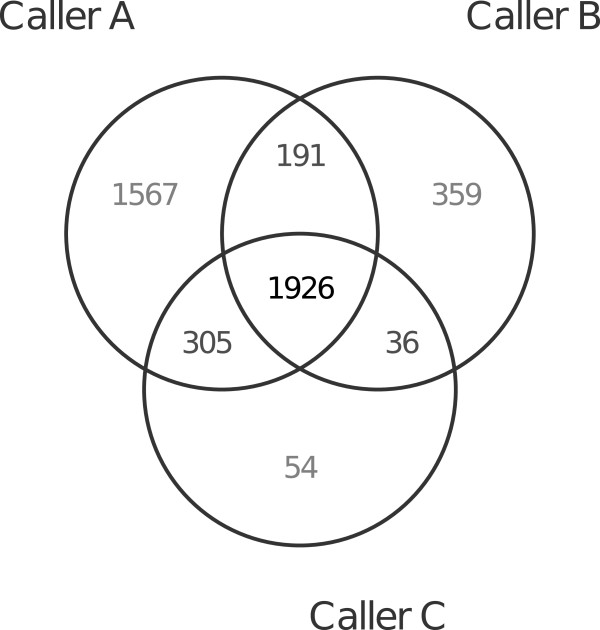
Venn diagram of the point mutations detected by three callers on 20 TCGA endometrial tumor-normal exome-seq pairs.

One natural and simple way to build a combined caller is to take intersections or unions of the calls from three callers as final calls. For example, one may take the mutations detected by all callers (ABC), or take intersections of mutations from two callers (AB, AC, or BC), or take calls detected by at least two callers (‘2orMore’), or even take calls detected by any caller (Union). This strategy is very intuitive and can be immediately used in practice once a Venn diagram is drawn from calls, as in Figure 1. Note that building this type of combined caller does not require a validation dataset — although estimating its performance does.

### Cumulatively adding mutation sets based on combination call status

We explained how the sets of mutation sites defined by a Venn diagram could be used to build a combined caller. Restricting ourselves to mutation sets corresponding to a combination of detection statuses of the *K* callers, we obtain a partition of the mutation sites into 2^*K*^-1 disjoint subsets. This partition can be used to systematically sort mutations by some measure of confidence that we have in their being somatic mutations. On Figure 1, these 2^3^-1=7 disjoint sets are ABC, AB without C, AC without B, BC without A, A only, B only, and C only. We sort these 2^*K*^-1 disjoint sets by their validation rate, *i.e.*, by the proportion of true mutations that they contain, as shown on Table 1. These sorted sets of sites define a sequence of combined callers, sorted by stringency. The most stringent combined caller only predicts the site in the first set to be mutations. Then less stringent combined callers can be defined by adding the sites in the sorted sets.

**Table 1:** **Validation results of the seven disjoint mutation sets shown in Figure **1

**Combination**	**Val.**	**FP**	**TP**	**cFP**	**cTP**
**call status**	**rate (%)**	**count**	**count**	**rate**	**rate**
All callers	99.4	12	1,914	1.2	55.3
Caller A and C only	96.4	11	294	2.4	63.8
Caller A and B only	96.3	7	184	3.1	69.1
Caller B and C only	94.4	2	34	3.3	70.1
Caller C only	79.6	11	43	4.4	71.3
Caller A only	59.7	632	935	69.1	98.4
Caller B only	15.9	302	57	100	100

For each mutation set (row), the validation rate (Val. rate), the false positive (FP) and true positive (TP) counts, and the cumulative false positive (cFP) and cumulative true positive (cTP) rates in percentage, are presented. Mutation sets are ordered by the validation rate.

### Fitting logistic models using the call status and genomic features

Stacked generalization was first introduced in the neural network community [12] and later adapted to the statistics literature [9], as a systematic way to combine classifiers.

Given a set of calls *c*_*i* *k*_∈{0,1} for site 1≤*i*≤*n* and caller 1≤*k*≤*K*, stacking aims at building a linear function of the calls for each site *i* which predicts its true status *y*_*i*_ as accurately as possible. In other words, we represent each site by its *K* calls from the different callers, and learn a new classifier of mutation sites in this feature space. Formally, given a set of *n* sites with known calls *c*_*i* *k*_ for all callers and known true status *y*_*i*_, a linear stacking approach would solve:

(1) $$\underset{\beta_{1},\ldots ,\beta_{K}}{\text{arg min}}\overset{n}{\underset{i=1}{\sum }}{\left(y_{i}-\overset{K}{\underset{k=1}{\sum }}\beta_{k}c_{\text{ik}}\right)}^{2},$$

*i.e.*, a linear regression in the call space, estimating weights *β*_*k*_ such that a linear combination of the calls based on these weights is close to the true mutation status. The mutation status of a new site *c*_*i*_ defined by its calls from the *K* individual callers would then be predicted via

(2) $$f(c_{i})\overset{\Delta }{=}\overset{K}{\underset{k=1}{\sum }}\beta_{k}c_{\text{ik}}.$$

In practice, we use a logistic model rather than a linear one, because it is better suited to binary classification [8] – we only have binary mutation status {0,1} as opposed to scores or continuous confidence measures. Our estimator therefore becomes:

(3) $$\underset{\beta_{1},\ldots ,\beta_{K}}{\text{arg min}}\overset{n}{\underset{i=1}{\sum }}\left\{\log\left(1+\exp\left\{\overset{K}{\underset{k=1}{\sum }}\beta_{k}c_{\text{ik}}\right\}\right)-y_{i}\overset{K}{\underset{k=1}{\sum }}\beta_{k}c_{\text{ik}}\right\}.$$

If the features *c*_*i* *k*_ are binary, which is the case if the individual callers returned binary decisions rather than continuous scores, the resulting classifier *f*(*c*_*i*_) is the sum of weights *β*_*k*_ for callers which classified the site *i* as a somatic mutation. It can only take 2^*K*^-1 distinct values on sites which were called by at least one caller. Each of these values corresponds to a unique combination of calls by the individual methods, which in turn corresponds to one of the disjoint subsets defined by the Venn diagram discussed in Section ‘Cumulatively adding mutation sets based on combination call status’. If the effects of callers are additive, then the ranking of the sites defined by *f* is expected to essentially behave like the more naive one defined in Section ‘Cumulatively adding mutation sets based on combination call status’.

The estimators defined by (1) and (3) combine the individual callers uniformly for all sites. It is however conceivable that some callers perform better for some types of sites, *e.g.*, those with low coverage, and less well for others. We now assume that some descriptors *g*_*i* *j*_, 1≤*j*≤*p*, of each site *i* are available besides the detection status of the three callers and the validation status. These descriptors could typically be genomic features.

Feature-weighted linear stacking (FWLS, [13]) replaces each parameter *β*_*k*_ of the stacking regression estimator (3) by a linear combination of the descriptors *g*_*i* *j*_:

(4) $$\beta_{k}=\overset{p}{\underset{j=1}{\sum }}\alpha_{\text{jk}}g_{\text{ij}},$$

where the *α*_*j* *k*_ parameters are weights corresponding to the relevance of feature *g*_*i* *j*_ to measure how predictive caller *k* is for site *i*. The weights *β*_*k*_ are therefore site-specific, accounting for the fact that the relevance *β*_*k*_ of a particular caller *k* may be different for sites with different genomic features.

Plugging weights (4) in the linear function (2) yields a different set of coefficients for each site *i* : $h(c_{i},g_{i})=\sum_{k=1}^{K}\beta_{k}c_{\text{ik}}=\sum_{k=1}^{K}\sum_{j=1}^{p}\alpha_{\text{jk}}g_{\text{ij}}c_{\text{ik}}$. *h* is now a linear function of the *K*×*p* products of features *g*_*i* *j*_ and calls *c*_*i* *k*_ so FWLS equivalently amounts to: 

• describing each site by this extended set of features, and

• estimating a linear classifier of mutation sites in this space.

Formally, after plugging (4) in our stacking estimator (3) we see that FWLS solves:

(5) $$\underset{\gamma_{1},\ldots ,\gamma_{K\times p}}{\text{arg min}}\overset{n}{\underset{i=1}{\sum }}\left\{\;\log\left(1+\exp\left\{\overset{K\times p}{\underset{l=1}{\sum }}\gamma_{l}x_{\text{il}}\right\}\;\right)-y_{i}\overset{K\times p}{\underset{l=1}{\sum }}\gamma_{l}x_{\text{il}}\right\},$$

where $x_{\text{il}}\in {\mathbb{R}}^{K\times p}$ contains all the products of calls and genomic features for site *i*. The *K*×*p* parameters *γ*_*l*_ are the weights of the logistic regression. They are strictly equivalent to the *α*_*j* *k*_ parameters of (4), we only use them to emphasize that FWLS can be formulated as a regular logistic regression estimator in an expanded feature space.

In the experiments of this paper, we consider all combinations of call status defined in Section ‘Cumulatively adding mutation sets based on combination call status’, *i.e.*, all products of single calls rather than the single calls. Technically this can still be cast as a FWLS model, by adding all single calls and products of single calls to the set of features *g*_*i* *j*_. In practice, our implementation relies on (5), *i.e.*, on a logistic regression in an expanded feature space.

Finally, since the resulting feature space can become large, we choose to use an *ℓ*_1_-penalized version of (5):

(6) $$\begin{array}{c} \underset{\gamma_{1},\ldots ,\gamma_{K\times p}}{\text{arg min}}\;\overset{n}{\underset{i=1}{\sum }}\left\{\;\log\;\left(\;1\;+\;\exp\left\{\;\overset{K\times p}{\underset{l=1}{\sum }}\gamma_{l}x_{\text{il}}\;\right\}\;\right)\;-\;y_{i}\;\;\overset{K\times p}{\underset{l=1}{\sum }}\gamma_{l}x_{\text{il}}\;\right\}\;+\;\lambda \;\;\overset{K\times p}{\underset{l=1}{\sum }}|\gamma_{l}|. \end{array}$$

Penalizing the *ℓ*_1_ norm $\overset{K\times p}{\underset{l=1}{\sum }}|\gamma_{l}|$ of the parameter is known to lead to sparse estimators [14], and λ∈ℝ is used to adjust the level of sparsity.

### Implementation and evaluation of combined callers

The approach of building a combined caller by taking intersections or unions (Section ‘Taking intersections or unions’) does not require a training set, and evaluation of the caller can be done straightforwardly on a test set. The approach that cumulatively adds disjoint subsets (Section ‘Cumulatively adding mutation sets based on combination call status’) uses a training set to determine the order of subsets (by computing the validation rate of each subset), and evaluates the performance on a test set using the order. For the approach building a caller by fitting a logistic model (Section ‘Fitting logistic models using the call status and genomic features’), a training set is used to estimate the *γ*_*l*_ parameters of (6). In order to choose the hyperparameter *λ*, we perform 10-fold cross validation on the training set for each candidate *λ* to estimate the error of the associated model. Then the most parsimonious model whose error is no more than one standard error above the error of the best model is chosen. Once *λ* is selected, we re-estimate *γ*_*l*_ using this *λ* on the whole training set, and evaluate its performance on the test set. Experiments were conducted using the R package glmnet [15], which implements penalized GLMs, in particular the *ℓ*_1_ penalized logistic regression of which (6) is an instance. The R scripts that contain our detailed implementation are included as Additional file 1.

## Results

We have used the mutation datasets generated for the TCGA endometrial study [10]. For 194 tumor-normal Illumina exome-sequence pairs, somatic-mutation calling was done by three centers whose algorithms are referred to here as Caller A, B, and C. In total, 51,648 single nucleotide variant (SNV) type of mutations were detected. A large fraction of the mutations were targeted for custom capture validation. As explained in the Additional file 2: Supplementary Methods, these sites were captured using the Nimblegen technology and then re-sequenced independently using an Illumina HighSeq 2000. In particular, impartial validation (i.e. validating all calls from all callers) was carried out for all mutations in (1) a randomly selected 20 patients and (2) an additional 243 genes of interest from the remaining 174 patients. Validation status was successfully determined for all but a small fraction (less than 5%) of the validated mutations. For more details about the validation and determining the validation status, see Additional file 2: Supplementary Methods. Our final dataset consists of the successfully validated mutations: (1) 4,438 sites in the selected 20 patients and (2) an additional 2,308 sites within the 243 genes of interest. Note that almost all of these sites (> 95%) are included as example datasets in our software package (Additional file 1).

For each point mutation site in our final dataset, we know the validation status (‘somatic’ or ‘non-somatic’), the call status (i.e., whether or not it was detected) by each of the three callers, the mutation substitution type (combination of the reference allele and the variant allele), and the sequencing depth and the variant allele fraction in each tumor and normal sample based on the exome sequence data that was used for mutation-calling. A brief summary of our dataset is included as Table 1, Additional file 2: Table S1 and Figures S1–S4. Caller B provided more information besides the positions of the detected mutations. For a broader set of somatic variants (candidate mutations), it reported the mutation quality score as well as the pass/fail status of individual filters at each site. Although the detailed description of each filter was not available, the filter outcomes were available (Additional file 2: Table S2), which we were able to use for improving Caller B’s performance (Section ‘Improving a single caller’s performance using details of its filters’). In Section ‘Building and evaluating combined callers’, we demonstrate how to build a combined caller using the calling status of the three individual mutation callers and a few genomic features. In Section ‘Improving a single caller’s performance using details of its filters’, we show the potential for improving the performance of an individual caller using more detailed outputs, using Caller B as an instance.

### Building and evaluating combined callers

We first used the mutations detected from the 20 selected patients (total: 4,438) to build and evaluate combined callers. Assuming (for illustrative purposes) that the characteristics of our mutations are not affected by sample-specific features, we randomly split the data into 50% training and 50% test sets. Other fractions were explored, but the qualitative conclusions were similar as long as there was enough data to train the model, e.g., more than 20% of the total.

**Figure 2:**
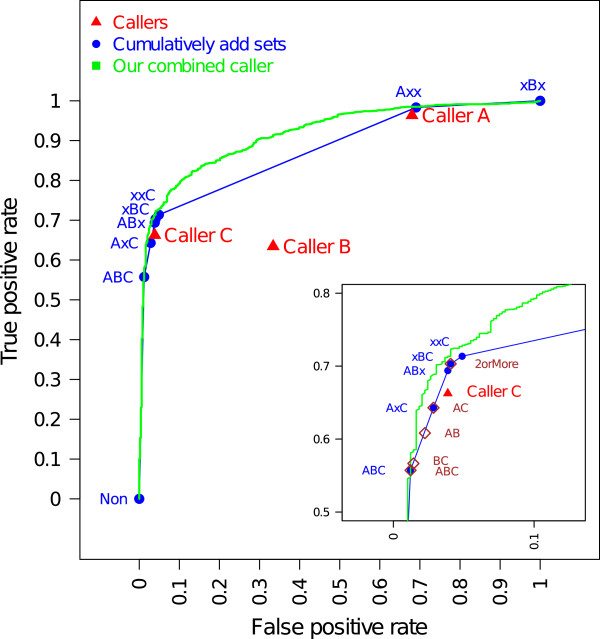
**Performances of individual and combined callers.** Model fitting was done using a random 50% of the point mutations detected from the selected 20 patients, and evaluation was done based on the remaining half. In the main panel, the true positive and the false positive rates of various callers are shown: (1) three individual callers (red filled triangles): Caller A, Caller B, and Caller C, (2) the caller that cumulatively adds mutation sets based on the combination call status in the order of the validation rate (connected blue dots), (3) the combined caller built by fitting a logistic model (for details, see text) (green line). The area near the point showing Caller C’s performance is enlarged and shown as a small sub-panel on the lower right part of the main figure. This panel further indicates the performance of the callers that take unions or intersections of calls from three callers (brown diamonds): all callers (ABC), intersections of two callers (AB, AC, or BC), called by more than two callers (‘2orMore’).

The performance of the combined caller constructed by fitting a logistic model (defined in Section ‘Fitting logistic models using the call status and genomic features’) is shown as a receiver operating characteristic (ROC) curve in Figure 2. The explanatory variables for this logistic model consist of the combination call status (7-1 variables), sequencing depth and variant allele fraction in each tumor and normal sample (4 variables), mutation substitution type (12-1 variables), and interactions between the combination call status variables and other features (90 variables). Note that we used combination call status (7-1 variables) instead of the call status of each individual caller (3 variables) as shown in (6) in Section ‘Fitting logistic models using the call status and genomic features’. We used the combination call status, since we do not want to assume that the effects of callers are necessarily additive. For example, in reality, a certain sequence feature may mislead two callers, but the remaining single caller may have a better filter for it. Therefore rather than imposing additivity, we would like to characterize each combination call status separately. The model fitting was done based on a randomly selected 50% training sites, then prediction was made on the remaining 50% test sites, enabling us to sort the mutations. A more stringent caller can be constructed by taking a smaller percentage of high-ranked mutations as final calls, and a more liberal caller can be constructed by including a larger percentage of mutations as final calls.

The performances of individual callers and combined callers are summarized in Figure 2. Note that validation was done only for the mutations that were detected by at least one of the three callers, and therefore, the union of all mutations comprises all true positives and all false positives. The results of three individual callers are given at three points with different false positive rates, i.e., different stringency levels. Caller A is the most liberal in the sense that it detected many false positives (FP rate at 68%) but also detected most of the true positives (TP rate at 96%). Caller C has a very small FP rate (4%) but detected only 67% of the true positives. Caller B performs poorer than Caller C, since it detected not only more false positives but also less true positives. The performance of the caller taking unions or intersections of the calls is marked as another set of points, inside of the sub-panel on the lower right part of the main panel. The stringency levels of these callers are not necessarily ordered. For example, the set of mutations called by two or more callers (2orMore) is nested within any intersection of two callers (AB, AC, or BC), but no ordering exists among the latter three intersections. In contrast to this, the performance of the caller adding mutations sets cumulatively is shown as a connected set of blue dots because of the natural ordering determined based on the validation rates. In reality, the ordering may not be the same between the training set and the test set. When the validation rates are very similar among the mutation subsets or the number of mutations in each set is very small, sampling variation could easily result in a different ordering. In the training set, the validation rates of the mutation set called by A and C but not B, and the set called by A and B but not C, are 97.99% and 97.96%, respectively.

Overall, our combined caller obtained by fitting a logistic model outperforms the individual callers and other naive combinations. The ROC curve of this combined caller is above of all the points representing the performance of individual callers, although sometimes only slightly so. Further, the combined caller allows us to assess the performance across the full range of stringency levels.

### Improving a single caller’s performance using details of its filters

For Caller B, mutation quality scores as well as the outcomes of individual filters were available for a broader set of somatic variants. (Note that for each caller, the detected mutations are the somatic variants that passed all the filters implemented by that caller.) In Figure 2, the performance of Caller B was shown as a single point. Here, we demonstrate how such extra details besides the call status can be used to improve the performance. Furthermore, to prove the validity of our approach in a wider dataset, we trained and tested on two different mutation datasets that were generated for the TCGA endometrial study using the same mutation calling algorithms, but constructed from different genomic regions as well as different tumor and normal samples. Specifically, we trained a model on the mutations from the 243 genes of interest from 174 patients (our second dataset described at the beginning of Section ‘Results’), then evaluated on the mutations from the whole exomes of the 20 patients (first dataset). A similar analysis was performed with the roles of the two datasets switched (Additional file 2: Figure S5).

**Figure 3:**
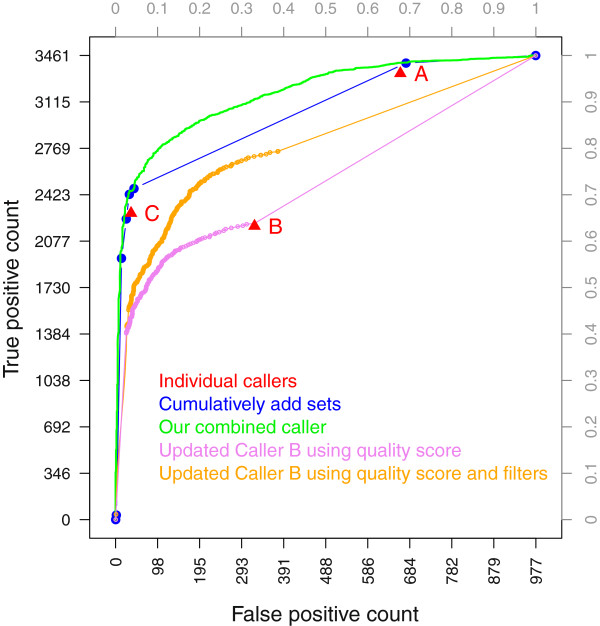
**ROC curve of an improved Caller B built by fitting a logistic model using the mutation quality score and individual filters of Caller B.** Model fitting was done using the point mutations in 243 genes of interest from 174 patients excluding the 20 patients, and evaluation was done on the point mutations in the 20 selected patients. The performances of three individual callers (red filled triangles), the combined caller that cumulatively adds mutation sets (connected blue dots), and the combined caller by fitting the logistic model (green lilne) are shown for comparison purposes. ROC curves of two updated versions of Caller B are shown. One version is obtained by ranking the mutations detected by Caller B using the mutation quality score of Caller B (violet line), and the other version by fitting a logistic model using the mutation quality score and the individual filters of Caller B on an extended set of mutations that were detected by at least one of the three callers (orange line).

Since a mutation quality score was available for Caller B, we first drew an ROC curve by sorting the calls that were detected by Caller B (Figure 3). As expected, the right most point in the ROC curve (besides the one at the FP rate of 1.0) corresponds to the point for which Caller B was previously evaluated. We then fitted a logistic model including the mutation quality score and the individual filter outcomes (indicator of pass/fail) from Caller B as explanatory variables. The estimated coefficients for the individual filters are summarized in Additional file 2: Table S2 (note that these coefficients were estimated from a set of ascertained sites for which each site was called by at least one of the three callers).

By utilizing the outcomes of individual filters, Caller B’s performance has improved substantially (Figure 3). At a false positive rate of 33%, the true positive rate increases from 63% to 78%, detecting 520 more mutations. This highlights the importance of having the full details of all features involved in the final decision on a variant.

Furthermore, if similar details were available for Caller A and C, then we could generalize the logistic model in previous section (Section ‘Building and evaluating combined callers’) including outcomes of individual filters from all callers, which potentially leads to a higher power as well as better insight on the cause of mutation-calling errors.

## Discussion

In this paper, we present an approach for effectively building a combined caller using the outputs from three mutation callers. Our approach is valid with more than three callers or less concordant mutation call outputs, as long as impartial validation data is available for all calls from all mutation callers as a training data, and the relative performance of individual callers is expected to be consistent between the training set and the test set. The combining approach could be even more beneficial if the individual callers agreed less — assuming (i) they all had comparable individual performances and (ii) the set of loci on which each caller is right could be characterized in terms of genomic features. In this case, the FWLS approach could learn the type of locus on which each caller is typically right and output the best answer for each new locus, resulting in a more accurate calling.

We have analyzed mutation sites that were successfully validated based on the criteria described in Additional file 2: Supplementary Methods. Those validation criteria may not be perfect, but we found them reasonable to demonstrate our approach. Changes in validation criteria can result changes in individual callers’ performances and thus the final model estimated. For example, more stringent criteria are likely to treat all very rare mutations as false calls, and thus in our exercise, may reduce the sensitivity of Caller A to a large extent. However, our approach remains to provide a convenient framework to build the best combined model, given any validation status. In practice, determining validation status based on an independent sequencing data can be very challenging, and developing highly accurate validation method itself is another research topic. Working on better validation is out of scope for our paper, but if uncertainty in the validation could be quantified, it could be used in the logistic model fitting to weight more accurate calls.

In practice, an effective validation strategy is essential for building a successful model. In principle, a training dataset is supposed to contain all sites characterizing a wider dataset for which one wishes to apply the estimated model. Therefore, a validation dataset needs to include enough sites to learn the behaviors of the mutation-calling algorithms across a broad spectrum of genomic features. Another important issue is to have impartially validated sites. If validation is done partially, then the composition of a training dataset is biased and thus the estimated parameters and the performance are also biased.

## Conclusions

Our approaches provide a unified framework for dealing with multiple somatic-mutation callers. If the callers provide only the list of positions detected as mutations, then it is difficult to compare them, or to investigate the tradeoff between the stringency of the calling-procedure and its power to detect true mutations. Our combined caller can be used to overcome these difficulties. It offers an evaluation of its performance across the full range as an ROC curve, and in addition, allows easy comparison with individual callers.

Furthermore, we have shown that it is feasible to build a combined caller that performs better than all the individual callers, one which could be better (even slightly) than a caller combining calls only based on the detection status. An even more powerful caller can possibly be built when more features associated with calling performance are available, such as individual details of the filters used by each caller or the measure of strand bias.

Finally, we demonstrate the potential for building a combined caller using a small validation dataset (generated for a subset of regions or samples in the original study), which can be applied to a wider dataset to assign a confidence measure that can be used for ranking the mutations from multiple callers. Our two mutation datasets, one from the selected 20 patients and the other from 243 genes of interest across 174 patients share protocols (sample preparation, sequencing technology, alignment methods, and the applied mutation-calling algorithms) but differ for genomic regions and the tumor and normal samples used for calling. The results from training the model using one of the datasets and evaluating on the other suggest that the estimated models based on these validation datasets are generally applicable to the mutations from whole exomes of all 194 endometrial patients.

## Abbreviations

TCGA: The cancer genome atlas; SNV: Single nucleotide variant; FWLS: Feature-weighted linear stacking; FP: False positive; FN: False negative; TP: True positive; ROC: Receiver operating characteristic.

## Competing interests

The authors declare that they have no competing interests.

## Authors’ contributions

SYK participated in the design of the study, carried out statistical analyses and drafted the manuscript. LJ participated in the design of the study, and drafted the manuscript. TPS conceived the study, participated in its design and helped to draft the manuscript. All authors read and approved the final manuscript.

## Supplementary Material

Additional file 1 **Software package.** A.tar.gz file that contains R scripts and example datasets to illustrate our approaches. The package also includes a manual file (pdf) explaining how to run the R scripts. Click here for file

Additional file 2 **Supplementary information.** A.pdf file including Supplementary Methods, Tables and Figures. Click here for file

## References

[B1] Hansen NF Gartner JJ Mei L Samuels Y Mullikin JC **Shimmer: detection of genetic alterations in tumors using next-generation sequence data** Bioinformatics 2013 29 12 1498 1503 10.1093/bioinformatics/btt183 23620360 PMC3673219

[B2] Cibulskis K Lawrence MS Carter SL Sivachenko A Jaffe D Sougnez C Gabriel S Meyerson M Lander ES Getz G **Sensitive detection of somatic point mutations in impure and heterogeneous cancer samples** Nat Biotechnol 2013 31 3 213 219 10.1038/nbt.2514 23396013 PMC3833702

[B3] Saunders CT Wong WS Swamy S Becq J Murray LJ Cheetham RK **Strelka: accurate somatic small-variant calling from sequenced tumor-normal sample pairs** Bioinformatics 2012 28 14 1811 1817 10.1093/bioinformatics/bts271 22581179

[B4] Ding J Bashashati A Roth A Oloumi A Tse K Zeng T Haffari G Hirst M Marra MA Condon A Aparicio S Shah SP **Feature-based classifiers for somatic mutation detection in tumour-normal paired sequencing data** Bioinformatics 2012 28 2 167 175 10.1093/bioinformatics/btr629 22084253 PMC3259434

[B5] Roth A Ding J Morin R Crisan A Ha G Giuliany R Bashashati A Hirst M Turashvili G Oloumi A Marra MA Aparicio S Shah SP **JointSNVMix: a probabilistic model for accurate detection of somatic mutations in normal/tumour paired next-generation sequencing data** Bioinformatics 2012 28 7 907 913 10.1093/bioinformatics/bts053 22285562 PMC3315723

[B6] Larson DE Harris CC Chen K Koboldt DC Abbott TE Dooling DJ Ley TJ Mardis ER Wilson RK Ding L **SomaticSniper: identification of somatic point mutations in whole genome sequencing data** Bioinformatics 2012 28 3 311 317 10.1093/bioinformatics/btr665 22155872 PMC3268238

[B7] Lower M Renard BY de Graaf J Wagner M Paret C Kneip C Tureci O Diken M Britten C Kreiter S Koslowski M Castle JC Sahin U **Confidence-based somatic mutation evaluation and prioritization** PLoS Comput Biol 2012 8 9 1002714 10.1371/journal.pcbi.1002714 PMC3459886 23028300

[B8] Hastie T Tibshirani R Friedman J The Elements of Statistical Learning 2009 New York: Springer

[B9] Breiman L **Stacked regressions** Mach Learn 1996 24 1 49 64

[B10] The Cancer Genome Atlas Research Network **Integrated genomic characterization of endometrial carcinoma** Nature 2013 497 7447 67 73 10.1038/nature12113 23636398 PMC3704730

[B11] Li H Handsaker B Wysoker A Fennell T Ruan J Homer N Marth G Abecasis G Durbin R **The Sequence Alignment/Map format and SAMtools** Bioinformatics 2009 25 16 2078 2079 10.1093/bioinformatics/btp352 19505943 PMC2723002

[B12] Wolpert DH **Stacked generalization** Neural Netw 1992 5 241 259 10.1016/S0893-6080(05)80023-1

[B13] Sill J Takács G Mackey L Lin D **Feature-weighted linear stacking** CoRR 2009 **abs/0911.0460**. [http://arxiv.org/abs/0911.0460]

[B14] Tibshirani R **Regression shrinkage and selection via the lasso** J R Stat Soc B 1996 58 1 267 288

[B15] Friedman J Hastie T Tibshirani R **Regularization paths for generalized linear models via coordinate descent** J Stat Softw 2010 33 1 1 22 20808728 PMC2929880

