# Supplementary material for: Combining calls from multiple somatic mutation-callers
Source: BMC Bioinformatics. 2014 May 21;15:154. doi: 10.1186/1471-2105-15-154 (PMC4035752; doi:10.1186/1471-2105-15-154)
Supplement: Additional file 2 — Supplementary information. A.pdf file including Supplementary Methods, Tables and Figures. [file 1471-2105-15-154-S2.pdf]

Supplementary information

Combining calls from multiple somatic mutation-callers

Su Yeon Kim[1*], Laurent Jacob[2] and Terence P. Speed[1,3*]

[1]Department of Statistics, University of California at Berkeley, Berkeley, CA 94720 USA

[2]Laboratoire de Biométrie et Biologie Evolutive, Université de Lyon, Université Lyon 1, CNRS, INRA, UMR5558,Villeurbanne, France

[3]Walter and Eliza Hall Institute of Medical Research and the University of Melbourne, Parkville, Victoria, Australia

[*] Corresponding authors

Email addresses:

SYK: suyeonkim08@gmail.com
TPS: terry@stat.berkeley.edu

Supplementary Methods

Validation of the mutations detected by three callers

Mutations to validate were first captured using 'Nimblegen's SeqCap EZ' liquid phase libraries [1], then sequenced on Illumina HighSeq 2000. Based on the sequence alignments, the validation status for each mutation was determined as follows. The status was successfully determined only for the sites that were successfully captured and are covered by more than 10 reads in both tumor and normal samples. It is 'somatic' if the tumor vaf $\geq 8\%$ and the normal vaf $< 3\%$, or the tumor vaf is between 0% and 8%, and the normal vaf is zero. It is 'unknown' if the tumor vaf is between 0% and 8%, and the normal vaf is between 0% and 3%. Otherwise, it is 'non-somatic'. The variants with 'unknown' status were omitted in our analyses.

Supplementary Tables

Table S1: Validation results of the seven disjoint mutation sets that comprise the 2,308 mutation sites detected in the 243 genes of interest from 174 patients. For each mutation set (row), the validation rate (Val. rate), the false positive (FP) and true positive (TP) counts, and the cumulative false positive (cFP) and cumulative true positive (cTP) rates in percentage, are presented. Mutation sets are ordered by the validation rate.

Combination call status	Val. rate (%)	FP count	TP count	cFPR	cTPR
Caller B and C only	100.0	0	25	0.0	1.3
All callers	99.3	9	1212	2.5	63.5
Caller A and C only	98.3	4	232	3.6	75.4
Caller A and B only	96.3	3	79	4.4	79.5
Caller C only	86.2	4	25	5.6	80.7
Caller A only	59.7	238	352	71.7	98.8
Caller B only	18.4	102	23	100.0	100.0

Table S2: Individual filters of Caller B. For each of the sites in our mutation dataset, Caller B either did not report that site as a candidate variant (filter information is missing) or reported the outcome of applying individual filters listed below. If a site passed all the filters, then it is detected as a somatic mutation and marked as 'PASS', otherwise, all the filters that failed were indicated. For example, a site that failed for both 'blq' and 'mc3' filters was marked as 'blq; mc3' in the output of Caller B. Caller B's performance can be improved by fitting a logistic model with the quality score of Caller B and these individual filters as explanatory variables on an extended mutation dataset compiled from three callers. The coefficients for the individual filters estimated from the set of mutations from the 20 patients are shown in the second column, and those estimated from the mutations from the 243 genes not including mutations from the 20 patients are shown in the third column. Note that when filter information is missing, we assume that the site fails for all individual filters and the mutation quality score is zero.

Filter name	Coefficient based 20 patients	Coefficient based on 243 genes
bldp	-0.675	0.000
blq	-0.000	-0.148
idl10	-0.000	-0.000
ma	-0.000	-0.000
mc3	-0.098	-0.830
pbias	-0.000	-0.000
sbias	-0.000	0.140
q40	1.439	1.036

Supplementary Figures

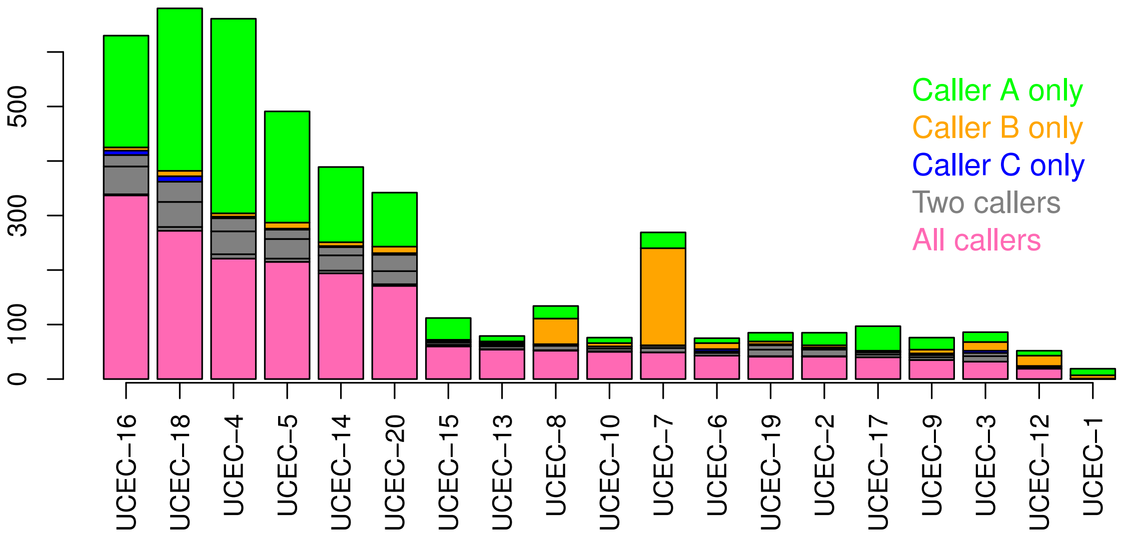

Figure S1: Counts of mutations across 20 selected patients that are classified based on the detection status of three callers: Caller A, B, and C. The patients (horizontal axis) are ordered by the number of the mutations detected by all callers. Notice that custom capture validation was not successful for one patient due to insufficient DNA materials, and thus only mutations from 19 patients are included in our final dataset.

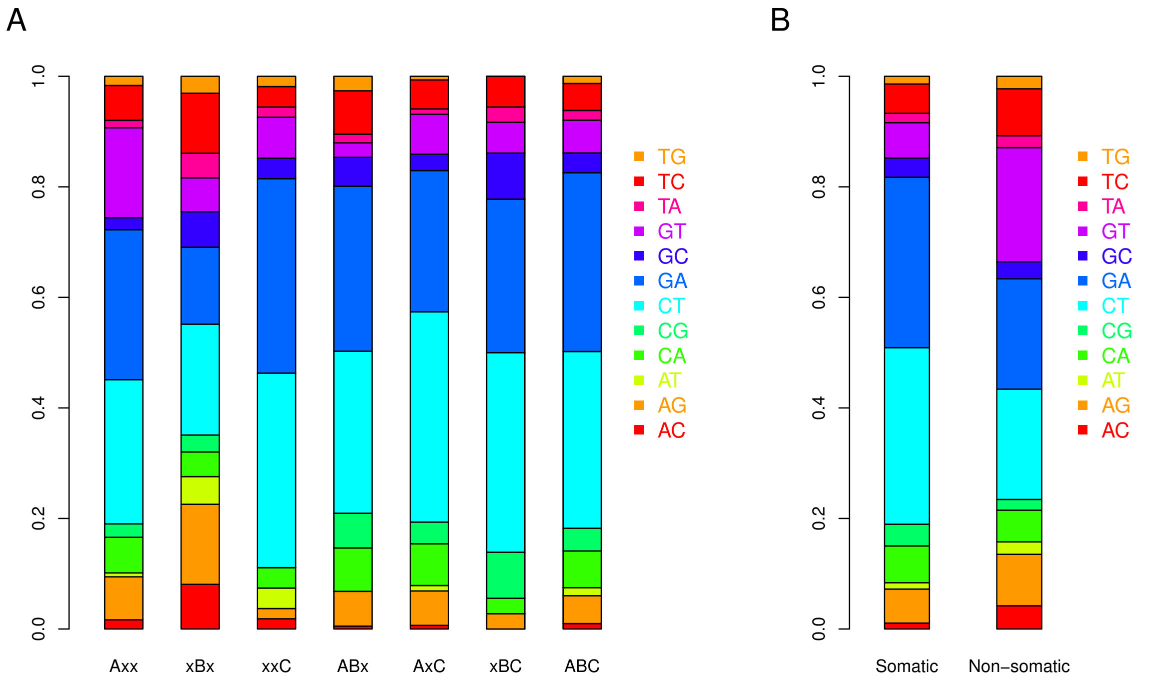

Figure S2: Distribution of the mutation substitution type (12 combinations of the reference allele and the variant allele). Mutations are classified either based on the combination call status (A) or based on the validation status (B). Mutations from the 20 selected patients are used.

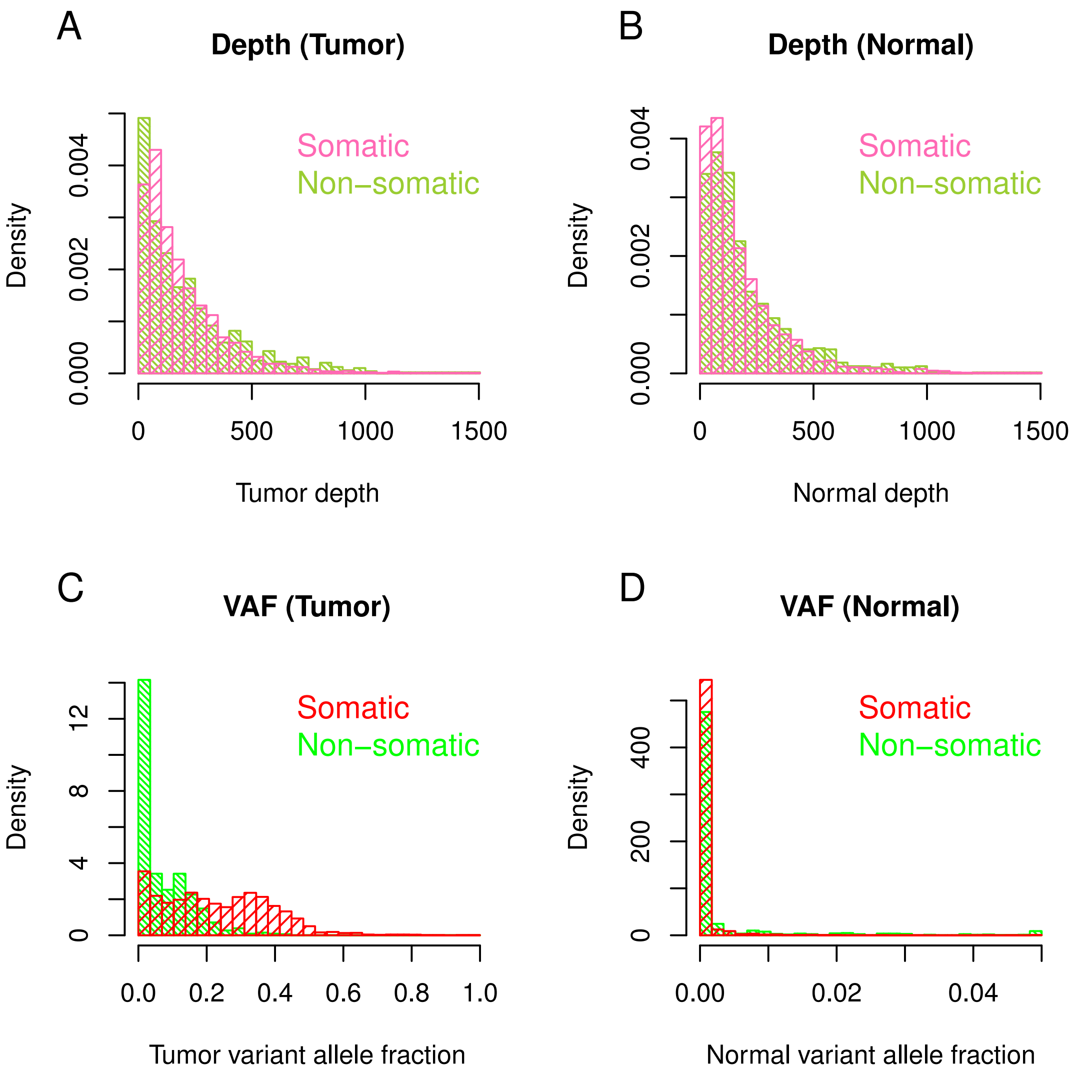

Figure S3: Comparison of the distribution of four genomic features between 'somatic' and 'non-somatic' mutations. The four features are the sequencing depth (upper row) and the variant allele fraction (lower row) in the tumor exome-seq (left column) or normal exome-seq (right column) data. Mutations from the 20 selected patients are used.

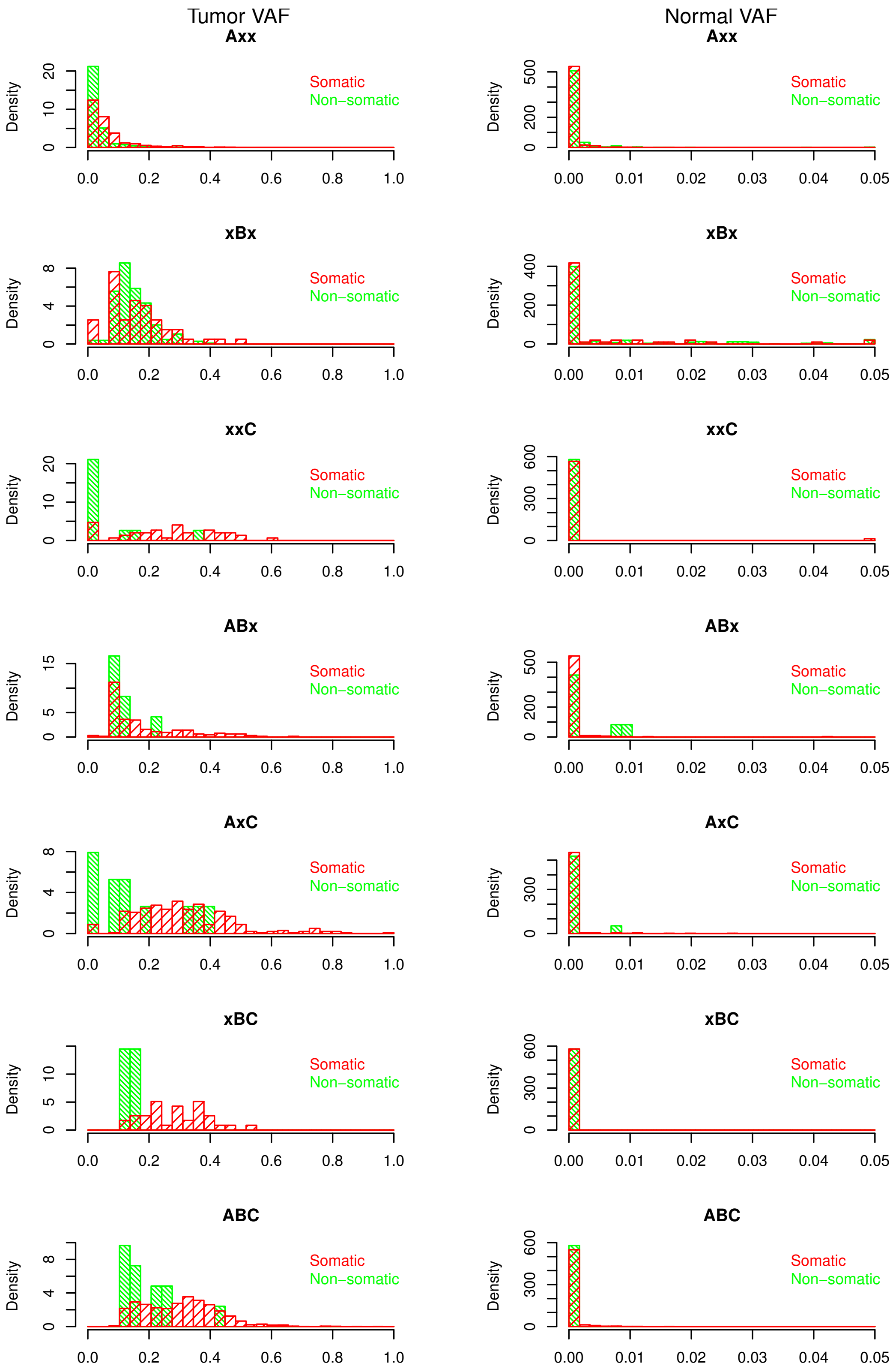

Figure S4: The distribution of the variant allele fraction (VAF) in the tumor exome-seq (left column) and the normal exome-seq (right column). Mutations are classified based on the combination call status (seven rows) and by the validation status (‘somatic’ as red, and ‘non-somatic’ as green). Mutations from the 20 selected patients are used.

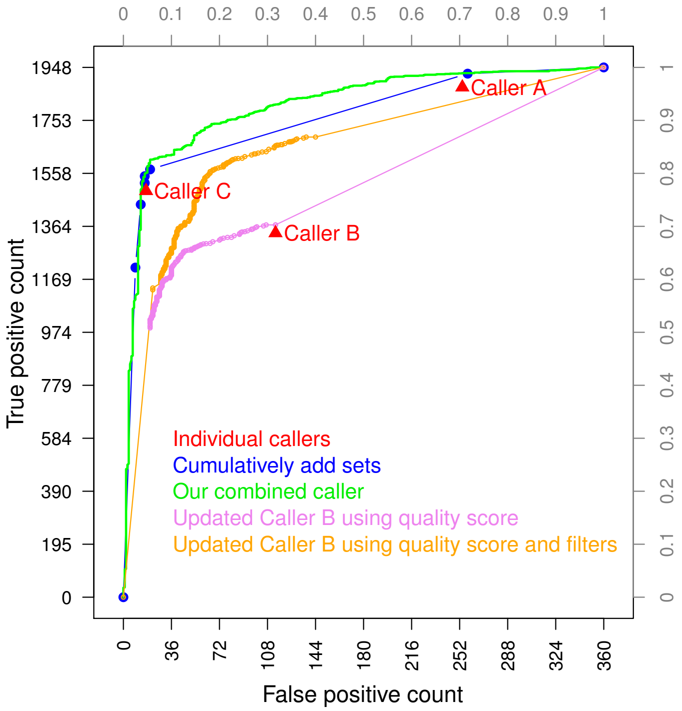

Figure S5: ROC curve of an improved Caller B built by fitting a logistic model using the mutation quality score and individual filters of Caller B. Model fitting was done using the mutations in the 20 selected patients, and evaluation was done on mutations in 243 genes of interest from 174 patients excluding the 20 patients. The performances of three individual callers (red filled triangles), the combined caller that cumulatively adds mutation sets (connected blue dots), and the combined caller by fitting the logistic model (green lilne) are shown for comparison purposes. ROC curves of two updated versions of Caller B are shown. One version is obtained by ranking the mutations detected by Caller B using the mutation quality score of Caller B (violet line), and the other version by fitting a logistic model using the mutation quality score and the individual filters of Caller B on an extended set of mutations that were detected by at least one of the three callers (orange line). Note that the same analysis was done as Figure 3 in the main text with the training and test datasets switched.

References

[1] Kiialainen, A., Karlberg, O., Ahlford, A., Sigurdsson, S., Lindblad-Toh, K., Syvanen, A.C.: Performance of microarray and liquid based capture methods for target enrichment for massively parallel sequencing and SNP discovery. PLoS ONE **6**(2), 16486 (2011)
